# (1*R*,2*S*,5*R*)-(–)-Menthyl (*S*)-2-(methoxy­carbonyl)­benzene­sulfinate

**DOI:** 10.1107/S1600536813009112

**Published:** 2013-04-13

**Authors:** Maria Altamura, Antonio Guidi, Loic Jierry, Paola Paoli, Patrizia Rossi

**Affiliations:** a Chemistry Department, Menarini Ricerche S.p.A., Via dei Sette Santi 3, I-50131 Firenze, Italy; b ICS, Université de Strasbourg, France; c Dipartimento di Ingegneria Industriale, University of Firenze, Via S. Marta 3, I-50139 Firenze, Italy

## Abstract

In the title chiral sulfinic acid ester, C_18_H_26_O_4_S, the cyclo­hexane ring of the menthyl fragment adopts a chair conformation. The mol­ecular shape is defined by the dihedral angle of 47.87 (8)° between the mean planes of the cyclo­hexane and benzene rings. In the crystal, mol­ecules related by the screw axis are connected into chains along [010] by weak C_ar_—H⋯O=S contacts.

## Related literature

For the synthesis of the title compound, see: Klunder & Sharpless (1987 ▶) and of chiral sulfoxides, see: Drabowicz *et al.* (1982 ▶); Solladié *et al.* (1987 ▶). For applications of menthol in synthetic chemistry, see Oertling *et al.* (2007 ▶). For structural studies of analogous chiral sulfinic acid esters, see: Mariz *et al.* (2010 ▶); Heinemann *et al.* (2007 ▶); Cherkaoui & Nicoud (1995 ▶).
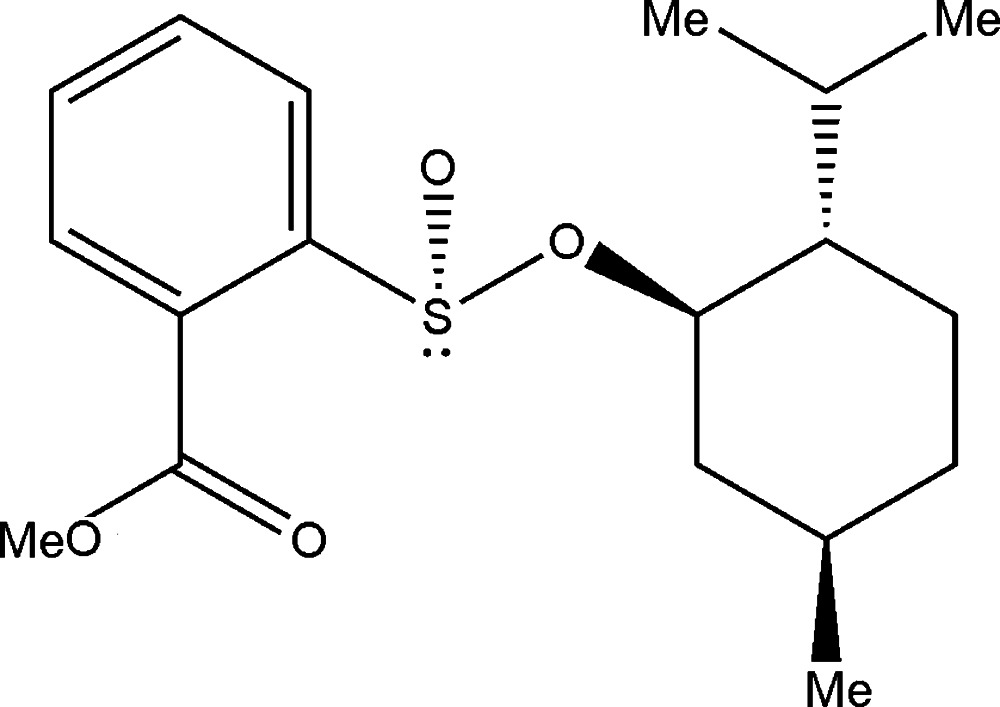


## Experimental

### 

#### Crystal data

C_18_H_26_O_4_S
*M* *_r_* = 338.45
Monoclinic, 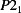

*a* = 9.7918 (2) Å
*b* = 9.3938 (2) Å
*c* = 10.6998 (2) Å
β = 112.176 (2)°
*V* = 911.39 (3) Å^3^
*Z* = 2
Cu *K*α radiation
μ = 1.72 mm^−1^
*T* = 150 K
0.26 × 0.22 × 0.08 mm

#### Data collection

Oxford Diffraction Xcalibur PX diffractometer
Absorption correction: multi-scan (*CrysAlis RED*; Oxford Diffraction, 2006 ▶) *T*_min_ = 0.660, *T*_max_ = 0.872
4681 measured reflections
2354 independent reflections
2115 reflections with *I* > 2σ(*I*)
*R*_int_ = 0.021

#### Refinement

*R*[*F* ^2^ > 2σ(*F* ^2^)] = 0.031
*wR*(*F* ^2^) = 0.078
*S* = 1.07
2354 reflections
209 parameters
1 restraint
H-atom parameters constrained
Δρ_max_ = 0.15 e Å^−3^
Δρ_min_ = −0.22 e Å^−3^
Absolute structure: Flack (1983 ▶), 767 Friedel pairs
Flack parameter: 0.038 (18)

### 

Data collection: *CrysAlis CCD* (Oxford Diffraction, 2006 ▶); cell refinement: *CrysAlis RED* (Oxford Diffraction, 2006 ▶); data reduction: *CrysAlis RED*; program(s) used to solve structure: *SIR97* (Altomare *et al.*, 1999 ▶); program(s) used to refine structure: *SHELXL97* (Sheldrick, 2008 ▶); molecular graphics: *ORTEP-3 for Windows* (Farrugia, 2012 ▶); software used to prepare material for publication: *PARST* (Nardelli, 1995 ▶).

## Supplementary Material

Crystal structure: contains datablock(s) I, global. DOI: 10.1107/S1600536813009112/yk2089sup1.cif

Structure factors: contains datablock(s) I. DOI: 10.1107/S1600536813009112/yk2089Isup2.hkl

Supplementary material file. DOI: 10.1107/S1600536813009112/yk2089Isup3.cml

Additional supplementary materials:  crystallographic information; 3D view; checkCIF report

## Figures and Tables

**Table 1:** Hydrogen-bond geometry (Å, °)

*D*—H⋯*A*	*D*—H	H⋯*A*	*D*⋯*A*	*D*—H⋯*A*
C5—H5⋯O4^i^	0.95	2.42	3.337 (3)	161

Symmetry code: (i) 
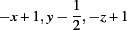
.

**Fig. 1.**
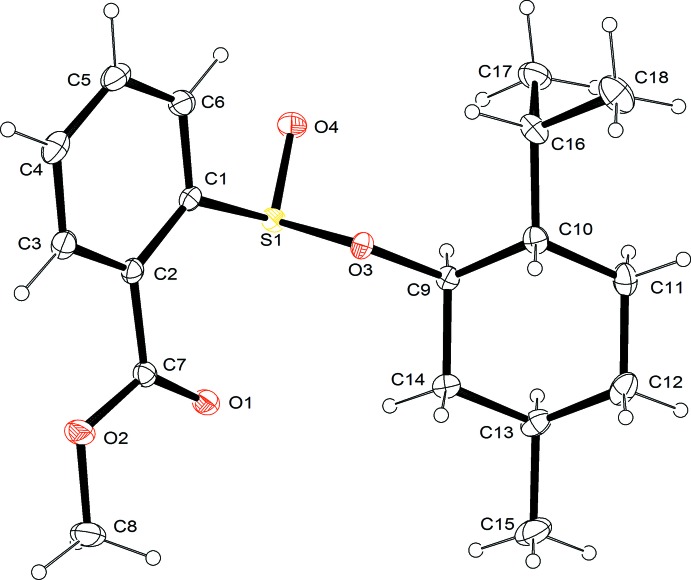
Crystal structure of the title compound with labelling and displacement ellipsoids drawn at the 50% probability level.

**Fig. 2.**
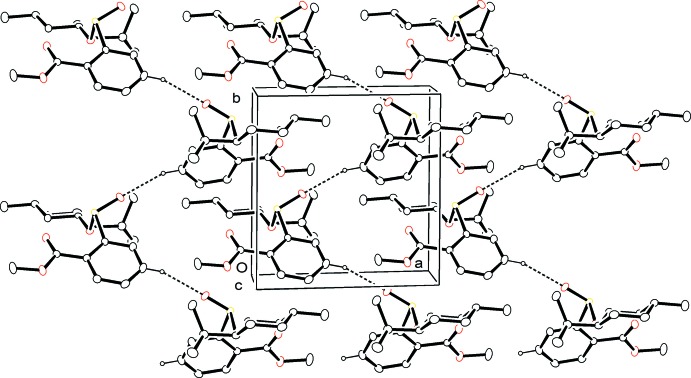
Crystal structure of the title compound with view along the c-axis. Intermolecular interactions are shown as dashed lines.

## supplementary crystallographic information

### Comment

As a result of the trigonal pyramidal stereochemistry which characterizes the sulfur atom in organic sulfinic acid esters and sulfoxides bearing two different substituents, these species are chiral. Chiral sulfoxides are key intermediates for asymmetric synthesis and can be obtained from the reaction of an organometallic reagent (*e.g.* a Grignard reagent) with a diastereomerically pure sulfinate ester of menthol (Drabowicz *et al.*, 1982). On the other hand, menthyl sulfinates can be prepared by reaction of menthol (Oertling *et al.*, 2007) either with sodium sulfinates (Solladié *et al.*, 1987) or with a sulfonyl chloride in the presence of trimethylphosphite as *in situ* reducing agent (Klunder & Sharpless, 1987). We used the latter method to prepare the chiral sulfinic acid ester, (1*R*,2S,5*R*)-(-)-menthyl (*S*)-2-carbomethoxybenzenesulfinate, here reported. The overall molecular shape of the title compound depends on the dihedral angle formed between the mean plane defined by the ring atoms of the menthyl and of the phenyl groupings (132.13°(8)). Bond distances and angles about the sulfur atom, as well as the orientation of the isopropyl chain with respect to the menthyl ring are in keeping with those already reported for this molecular fragment (Heinemann *et al.*, 2007; Mariz *et al.*, 2010; Cherkaoui & Nicoud, 1995). In the crystal, molecules are connected *via* weak C_ar_—H···O contacts involving the double bonded oxygen atom of the sulfinate group as acceptor. The resulting molecular chain propagates along the *b* axis direction around the screw axis.

### Experimental

For the synthesis of the title compound, see: Klunder & Sharpless (1987). Crystals of the title compound suitable for single-crystal X-ray diffraction analysis were obtained by slow evaporation from a diethyl ether solution of the sulfinate ester.

### Refinement

All the H atoms were positioned with idealized geometry using a riding model and refined with *U*_iso_(H) 1.2 times *U*_eq_(C) (1.5 for methyl H atoms).

### Crystal data

**Table:**

C_18_H_26_O_4_S	*F*(000) = 364
*M**_r_* = 338.45	*D*_x_ = 1.233 Mg m^−^^3^
Monoclinic, *P*2_1_	Cu *K*α radiation, λ = 1.5418 Å
*a* = 9.7918 (2) Å	Cell parameters from 3618 reflections
*b* = 9.3938 (2) Å	θ = 4.5–64.6°
*c* = 10.6998 (2) Å	µ = 1.72 mm^−^^1^
β = 112.176 (2)°	*T* = 150 K
*V* = 911.39 (3) Å^3^	Platelet, colourless
*Z* = 2	0.26 × 0.22 × 0.08 mm

### Data collection

**Table:**

Oxford Diffraction Xcalibur PX diffractometer	2354 independent reflections
Radiation source: Enhance (Cu) X-ray Source	2115 reflections with *I* > 2σ(*I*)
Graphite monochromator	*R*_int_ = 0.021
Detector resolution: 8.1241 pixels mm^-1^	θ_max_ = 64.8°, θ_min_ = 4.5°
ω scans	*h* = −11→10
Absorption correction: multi-scan (*CrysAlis RED*; Oxford Diffraction, 2006)	*k* = −10→8
*T*_min_ = 0.660, *T*_max_ = 0.872	*l* = −11→12
4681 measured reflections	

### Refinement

**Table:**

Refinement on *F*^2^	Secondary atom site location: difference Fourier map
Least-squares matrix: full	Hydrogen site location: inferred from neighbouring sites
*R*[*F*^2^ > 2σ(*F*^2^)] = 0.031	H-atom parameters constrained
*wR*(*F*^2^) = 0.078	*w* = 1/[σ^2^(*F*_o_^2^) + (0.0533*P*)^2^] where *P* = (*F*_o_^2^ + 2*F*_c_^2^)/3
*S* = 1.07	(Δ/σ)_max_ < 0.001
2354 reflections	Δρ_max_ = 0.15 e Å^−^^3^
209 parameters	Δρ_min_ = −0.22 e Å^−^^3^
1 restraint	Absolute structure: Flack (1983), 767 Friedel pairs
Primary atom site location: structure-invariant direct methods	Flack parameter: 0.038 (18)

### Special details

**Table:**

Geometry. All s.u.'s (except the s.u. in the dihedral angle between two l.s. planes) are estimated using the full covariance matrix. The cell s.u.'s are taken into account individually in the estimation of s.u.'s in distances, angles and torsion angles; correlations between s.u.'s in cell parameters are only used when they are defined by crystal symmetry. An approximate (isotropic) treatment of cell s.u.'s is used for estimating s.u.'s involving l.s. planes.
Refinement. Refinement of *F*^2^ against ALL reflections. The weighted *R*-factor *wR* and goodness of fit *S* are based on *F*^2^, conventional *R*-factors *R* are based on *F*, with *F* set to zero for negative *F*^2^. The threshold expression of *F*^2^ > 2σ(*F*^2^) is used only for calculating *R*-factors(gt) *etc*. and is not relevant to the choice of reflections for refinement. *R*-factors based on *F*^2^ are statistically about twice as large as those based on *F*, and *R*- factors based on ALL data will be even larger.

### Fractional atomic coordinates and isotropic or equivalent isotropic displacement parameters (Å^2^)

**Table:**

	*x*	*y*	*z*	*U*_iso_*/*U*_eq_	
S1	0.13357 (6)	0.36878 (7)	0.29567 (5)	0.02816 (16)	
O1	−0.12928 (17)	0.2413 (2)	0.29271 (17)	0.0370 (5)	
O2	−0.14622 (19)	0.0826 (2)	0.44178 (18)	0.0456 (5)	
O3	0.10664 (16)	0.26365 (17)	0.16750 (14)	0.0276 (4)	
O4	0.27015 (18)	0.4479 (2)	0.31899 (17)	0.0393 (5)	
C1	0.1889 (2)	0.2232 (3)	0.4157 (2)	0.0276 (6)	
C2	0.0910 (3)	0.1423 (3)	0.4543 (2)	0.0278 (5)	
C3	0.1479 (3)	0.0401 (3)	0.5550 (2)	0.0351 (6)	
H3	0.0830	−0.0130	0.5844	0.042*	
C4	0.2987 (3)	0.0150 (3)	0.6128 (2)	0.0401 (7)	
H4	0.3364	−0.0556	0.6808	0.048*	
C5	0.3939 (3)	0.0927 (3)	0.5715 (2)	0.0417 (7)	
H5	0.4969	0.0741	0.6096	0.050*	
C6	0.3395 (3)	0.1976 (3)	0.4747 (2)	0.0350 (6)	
H6	0.4056	0.2526	0.4484	0.042*	
C7	−0.0714 (2)	0.1625 (3)	0.3865 (2)	0.0288 (5)	
C8	−0.3054 (3)	0.0939 (4)	0.3802 (3)	0.0586 (9)	
H8A	−0.3501	0.0279	0.4248	0.088*	
H8B	−0.3386	0.0700	0.2842	0.088*	
H8C	−0.3353	0.1915	0.3901	0.088*	
C9	0.0431 (2)	0.3366 (2)	0.03670 (19)	0.0252 (5)	
H9	0.0707	0.4396	0.0496	0.030*	
C10	0.1105 (3)	0.2704 (3)	−0.0566 (2)	0.0322 (6)	
H10	0.0819	0.1676	−0.0660	0.039*	
C11	0.0369 (3)	0.3372 (3)	−0.1967 (2)	0.0404 (7)	
H11A	0.0642	0.4391	−0.1920	0.048*	
H11B	0.0742	0.2898	−0.2602	0.048*	
C12	−0.1312 (3)	0.3239 (3)	−0.2505 (2)	0.0439 (7)	
H12A	−0.1740	0.3709	−0.3397	0.053*	
H12B	−0.1588	0.2220	−0.2628	0.053*	
C13	−0.1943 (3)	0.3903 (3)	−0.1564 (2)	0.0377 (7)	
H13	−0.1691	0.4939	−0.1486	0.045*	
C14	−0.1233 (2)	0.3239 (3)	−0.0165 (2)	0.0333 (6)	
H14A	−0.1609	0.3721	0.0464	0.040*	
H14B	−0.1511	0.2222	−0.0210	0.040*	
C15	−0.3628 (3)	0.3767 (5)	−0.2088 (3)	0.0574 (8)	
H15A	−0.3992	0.4212	−0.1445	0.086*	
H15B	−0.3903	0.2757	−0.2188	0.086*	
H15C	−0.4067	0.4242	−0.2964	0.086*	
C16	0.2812 (3)	0.2752 (3)	0.0022 (3)	0.0397 (7)	
H16	0.3151	0.2284	0.0928	0.048*	
C17	0.3439 (3)	0.4256 (3)	0.0237 (3)	0.0487 (8)	
H17A	0.3020	0.4796	0.0791	0.073*	
H17B	0.3188	0.4728	−0.0639	0.073*	
H17C	0.4514	0.4214	0.0697	0.073*	
C18	0.3433 (4)	0.1894 (4)	−0.0842 (3)	0.0599 (9)	
H18A	0.3017	0.0932	−0.0970	0.090*	
H18B	0.4509	0.1839	−0.0393	0.090*	
H18C	0.3176	0.2358	−0.1722	0.090*	

### Atomic displacement parameters (Å^2^)

**Table:**

	*U*^11^	*U*^22^	*U*^33^	*U*^12^	*U*^13^	*U*^23^
S1	0.0300 (3)	0.0257 (3)	0.0306 (3)	−0.0036 (3)	0.0135 (2)	−0.0045 (3)
O1	0.0298 (9)	0.0443 (12)	0.0374 (9)	0.0010 (8)	0.0133 (8)	0.0100 (9)
O2	0.0312 (9)	0.0591 (14)	0.0451 (10)	−0.0078 (9)	0.0127 (9)	0.0180 (10)
O3	0.0333 (8)	0.0240 (9)	0.0245 (8)	0.0019 (7)	0.0099 (7)	−0.0007 (7)
O4	0.0361 (9)	0.0407 (11)	0.0410 (9)	−0.0146 (9)	0.0144 (8)	−0.0045 (9)
C1	0.0289 (12)	0.0302 (15)	0.0209 (11)	−0.0024 (11)	0.0065 (10)	−0.0045 (10)
C2	0.0302 (12)	0.0306 (15)	0.0225 (11)	0.0002 (11)	0.0097 (9)	−0.0035 (10)
C3	0.0395 (14)	0.0363 (16)	0.0276 (12)	−0.0029 (12)	0.0104 (12)	−0.0004 (12)
C4	0.0456 (16)	0.0387 (18)	0.0291 (13)	0.0101 (13)	0.0062 (13)	0.0005 (12)
C5	0.0318 (14)	0.0530 (19)	0.0331 (13)	0.0105 (13)	0.0040 (12)	−0.0082 (14)
C6	0.0303 (13)	0.0414 (17)	0.0332 (13)	0.0015 (12)	0.0119 (11)	−0.0065 (12)
C7	0.0310 (13)	0.0292 (15)	0.0279 (12)	−0.0023 (11)	0.0131 (11)	−0.0033 (12)
C8	0.0299 (14)	0.083 (3)	0.0579 (18)	−0.0111 (16)	0.0109 (14)	0.0230 (18)
C9	0.0300 (11)	0.0190 (15)	0.0264 (11)	−0.0018 (10)	0.0102 (9)	0.0021 (10)
C10	0.0383 (13)	0.0275 (15)	0.0333 (12)	−0.0012 (12)	0.0163 (11)	0.0009 (11)
C11	0.0554 (16)	0.038 (2)	0.0332 (12)	−0.0040 (13)	0.0226 (12)	0.0031 (12)
C12	0.0538 (16)	0.0390 (18)	0.0301 (12)	−0.0056 (13)	0.0058 (11)	0.0043 (12)
C13	0.0370 (13)	0.0301 (18)	0.0368 (12)	−0.0022 (12)	0.0034 (11)	0.0035 (13)
C14	0.0284 (12)	0.0338 (17)	0.0352 (12)	−0.0003 (11)	0.0092 (10)	0.0000 (11)
C15	0.0369 (13)	0.068 (2)	0.0492 (15)	−0.0022 (17)	−0.0037 (12)	0.0096 (18)
C16	0.0421 (15)	0.0395 (18)	0.0469 (15)	0.0020 (13)	0.0275 (12)	0.0043 (14)
C17	0.0396 (15)	0.053 (2)	0.0575 (18)	−0.0113 (14)	0.0229 (14)	−0.0056 (15)
C18	0.065 (2)	0.056 (2)	0.076 (2)	0.0083 (18)	0.0462 (17)	−0.0065 (19)

### Geometric parameters (Å, º)

**Table:**

S1—O4	1.4669 (17)	C10—C16	1.548 (3)
S1—O3	1.6287 (16)	C10—H10	1.0000
S1—C1	1.813 (2)	C11—C12	1.530 (3)
O1—C7	1.203 (3)	C11—H11A	0.9900
O2—C7	1.333 (3)	C11—H11B	0.9900
O2—C8	1.449 (3)	C12—C13	1.501 (4)
O3—C9	1.469 (2)	C12—H12A	0.9900
C1—C6	1.388 (3)	C12—H12B	0.9900
C1—C2	1.403 (3)	C13—C14	1.525 (3)
C2—C3	1.392 (4)	C13—C15	1.535 (3)
C2—C7	1.490 (3)	C13—H13	1.0000
C3—C4	1.389 (4)	C14—H14A	0.9900
C3—H3	0.9500	C14—H14B	0.9900
C4—C5	1.382 (4)	C15—H15A	0.9800
C4—H4	0.9500	C15—H15B	0.9800
C5—C6	1.382 (4)	C15—H15C	0.9800
C5—H5	0.9500	C16—C18	1.517 (4)
C6—H6	0.9500	C16—C17	1.524 (4)
C8—H8A	0.9800	C16—H16	1.0000
C8—H8B	0.9800	C17—H17A	0.9800
C8—H8C	0.9800	C17—H17B	0.9800
C9—C14	1.514 (3)	C17—H17C	0.9800
C9—C10	1.522 (3)	C18—H18A	0.9800
C9—H9	1.0000	C18—H18B	0.9800
C10—C11	1.532 (3)	C18—H18C	0.9800
			
O4—S1—O3	107.34 (9)	C10—C11—H11A	109.2
O4—S1—C1	104.61 (10)	C12—C11—H11B	109.2
O3—S1—C1	92.81 (9)	C10—C11—H11B	109.2
C7—O2—C8	115.7 (2)	H11A—C11—H11B	107.9
C9—O3—S1	113.26 (13)	C13—C12—C11	111.8 (2)
C6—C1—C2	119.9 (2)	C13—C12—H12A	109.3
C6—C1—S1	115.80 (19)	C11—C12—H12A	109.3
C2—C1—S1	124.25 (17)	C13—C12—H12B	109.3
C3—C2—C1	118.9 (2)	C11—C12—H12B	109.3
C3—C2—C7	120.4 (2)	H12A—C12—H12B	107.9
C1—C2—C7	120.7 (2)	C12—C13—C14	109.7 (2)
C4—C3—C2	120.6 (2)	C12—C13—C15	112.2 (2)
C4—C3—H3	119.7	C14—C13—C15	110.7 (2)
C2—C3—H3	119.7	C12—C13—H13	108.0
C5—C4—C3	120.0 (3)	C14—C13—H13	108.0
C5—C4—H4	120.0	C15—C13—H13	108.0
C3—C4—H4	120.0	C9—C14—C13	111.34 (19)
C4—C5—C6	120.0 (2)	C9—C14—H14A	109.4
C4—C5—H5	120.0	C13—C14—H14A	109.4
C6—C5—H5	120.0	C9—C14—H14B	109.4
C5—C6—C1	120.5 (2)	C13—C14—H14B	109.4
C5—C6—H6	119.8	H14A—C14—H14B	108.0
C1—C6—H6	119.8	C13—C15—H15A	109.5
O1—C7—O2	123.5 (2)	C13—C15—H15B	109.5
O1—C7—C2	124.4 (2)	H15A—C15—H15B	109.5
O2—C7—C2	112.1 (2)	C13—C15—H15C	109.5
O2—C8—H8A	109.5	H15A—C15—H15C	109.5
O2—C8—H8B	109.5	H15B—C15—H15C	109.5
H8A—C8—H8B	109.5	C18—C16—C17	110.6 (2)
O2—C8—H8C	109.5	C18—C16—C10	110.9 (2)
H8A—C8—H8C	109.5	C17—C16—C10	113.6 (2)
H8B—C8—H8C	109.5	C18—C16—H16	107.1
O3—C9—C14	109.29 (17)	C17—C16—H16	107.1
O3—C9—C10	107.54 (18)	C10—C16—H16	107.1
C14—C9—C10	113.12 (18)	C16—C17—H17A	109.5
O3—C9—H9	108.9	C16—C17—H17B	109.5
C14—C9—H9	108.9	H17A—C17—H17B	109.5
C10—C9—H9	108.9	C16—C17—H17C	109.5
C9—C10—C11	108.34 (19)	H17A—C17—H17C	109.5
C9—C10—C16	112.98 (19)	H17B—C17—H17C	109.5
C11—C10—C16	115.0 (2)	C16—C18—H18A	109.5
C9—C10—H10	106.7	C16—C18—H18B	109.5
C11—C10—H10	106.7	H18A—C18—H18B	109.5
C16—C10—H10	106.7	C16—C18—H18C	109.5
C12—C11—C10	112.09 (19)	H18A—C18—H18C	109.5
C12—C11—H11A	109.2	H18B—C18—H18C	109.5

### Hydrogen-bond geometry (Å, º)

**Table:**

*D*—H···*A*	*D*—H	H···*A*	*D*···*A*	*D*—H···*A*
C5—H5···O4^i^	0.95	2.42	3.337 (3)	161

Symmetry code: (i) −*x*+1, *y*−1/2, −*z*+1.
